# Quickly Updatable Hologram Images Using Poly(*N*-vinyl Carbazole) (PVCz) Photorefractive Polymer Composite 

**DOI:** 10.3390/ma5081477

**Published:** 2012-08-22

**Authors:** Naoto Tsutsumi, Kenji Kinashi, Asato Nonomura, Wataru Sakai

**Affiliations:** Department of Macromolecular Science and Engineering, Kyoto Institute of Technology, Matsugasaki, Sakyo, Kyoto 606-8585, Japan; E-Mails: kinashi@kit.ac.jp (K.K.); na0352sh@yahoo.co.jp (A.N.); wsakai@kit.ac.jp (W.S.)

**Keywords:** photorefractive polymer, 3D holographic display, poly(*N*-vinyl carbazole) composite

## Abstract

Quickly updatable hologram images using photorefractive (PR) polymer composite based on poly(*N*-vinyl carbazole) (PVCz) is presented. PVCz is one of the pioneer materials of photoconductive polymers. PR polymer composite consists of 44 wt % of PVCz, 35 wt % of 4-azacycloheptylbenzylidene-malonitrile (7-DCST) as a nonlinear optical dye, 20 wt % of carbazolylethylpropionate (CzEPA) as a photoconductive plasticizer and 1 wt % of 2,4,7-trinitro-9-fluorenone (TNF) as a sensitizer. PR composite gives high diffraction efficiency of 68% at E = 45 V μm^−1^. Response speed of optical diffraction is the key parameter for real-time 3D holographic display. The key parameter for obtaining quickly updatable holographic images is to control the glass transition temperature lower enough to enhance chromophore orientation. Object image of the reflected coin surface recorded with reference beam at 532 nm (green beam) in the PR polymer composite is simultaneously reconstructed using a red probe beam at 642 nm. Instead of using a coin object, an object image produced by a computer was displayed on a spatial light modulator (SLM) and used for the hologram. The reflected object beam from an SLM was interfered with a reference beam on PR polymer composite to record a hologram and simultaneously reconstructed by a red probe beam.

## 1. Introduction 

Three dimensional (3D) visions are natural for human beings. Several technologies, such as anaglyph, polarization, field-sequential and recent 3D HDTV have been developed to reproduce 3D imaging by means of stereoscopy. These technologies are based on the synthesis of two images in the brain, one image is for the right eye and the other image is for the left eye. For this purpose, the special eyewear to be worn by the observer is required to reproduce the 3D image. On the other hand, holography produces real 3D images without wearing any special eye glasses. Holography was first invented by Gabor [1] in 1948. Holography is a unique technique to precisely reproduce and display 3D objects recorded in the media. In the security fields, holography is an important technology. The holographic art has attracted people. Conventional holograms are permanently recorded in the media such as silver halides, photopolymers, and dichromated gelatin, *etc*. After recording in the media and processing via the several steps required, these holograms are reconstructed with coherent light such as laser or incoherent light sources of LEDs. These media lack the capability of image-updating, resulting in a limitation of their use. In contrast, photorefractive (PR) polymers [2,3,4,5] are a promising media for providing updatable or rewritable holograms. Thus, using PR polymers, updatable real-time three-dimensional holograms can be recorded and simultaneously reconstructed or replayed. Recently Peyghambarian’s group developed updatable holographic displays using large area photorefractive polymers based on PATPD polymer [6,7,8] and the stereographic technique. We have demonstrated two dimensional images were recorded in the photorefractive polymers and simultaneously reconstructed using a probe beam [9]. Without applying external electric field, real-time holographic recording and simultaneous reconstructing was demonstrated [10]. Furthermore, there are several reviews and papers concerned with photorefractive characterization. Review articles of photorefractive materials [11] and photorefractive 3D display [12] is presented. Alq(3) improves the response time, efficiency, and breakdown voltage without a significant increase in absorption or loss of phase stability [13]. Using interdigitated coplanar electrodes, enhanced photorefractive response was reported [14]. Monolithic photorefractive composite with good phase stability (anti-phase separation) has been reported [15]. High response photorefractive polymer has also been reported [16]. Here, we present on a quickly rewritable or updatable holographic 3D display using photorefractive polymers with low glass transition temperature (T_g_). In this report, we present quickly updatable hologram images using a high performance PR polymer composite based on PVCz. PVCz photorefractive composite gives a high diffraction efficiency of 68% with fast response speed. Object images of reflected coin surface and reflected image on a spatial light modulator (SLM) were recorded with reference beam at 532 nm (green beam) in the PR polymer, and simultaneously reconstructed using a red probe beam at 642 nm. 

## 2. Experimental Section

### 2.1. Materials

**Figure 1:**
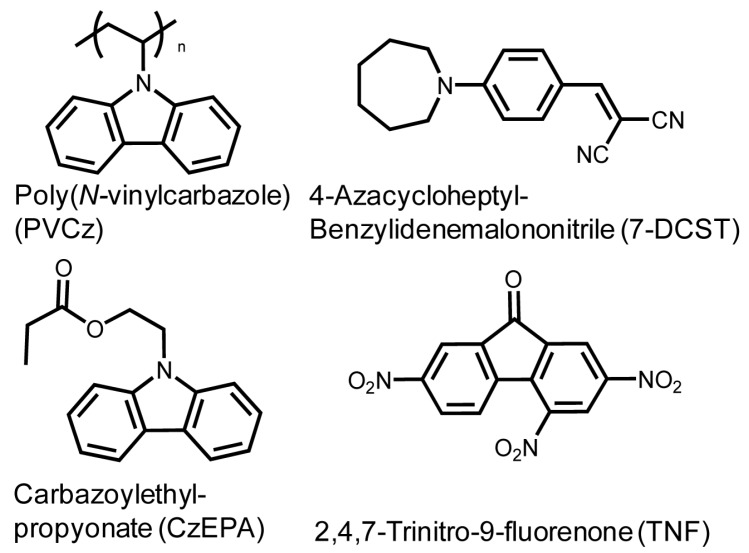
Structural formulae of poly(*N*-vinyl carbazole) (PVCz), 4-azacycloheptylbenzylidene-malonitrile (7-DCST), carbazolylethylpropionate (CzEPA), and 2,4,7-trinitro-9-fluorenone (TNF).

Photorefractive polymers are required photoconductivity and second order optical nonlinearity. To enhance the response speed of holographic imaging, plasticizer plays an important role to lower T_g_ of matrix close or below an operating temperature. Figure 1 shows the structural formulae of materials used. PVCz is a photoconductive polymer. 4-Azacycloheptylbenzylidene-malonitrile (7-DCST) is a second order optical nonlinear dye. 2,4,7-Trinitro-9-fluorenone (TNF) is used as a sensitizer to enhance the charge carrier generation. Carbazolylethylpropionate (CzEPA) is used as a photoconductive plasticizer to lower T_g_. Composite films consisted of a given weight ratio of PVCz, 7-DCST, CzEPA and TNF (44/35/20/1 by wt) were cast on a hot plate at 70 °C for 24 h followed by drying in a vacuum oven at 60 °C for 24 h. The obtained composite films were sandwiched between indium tin oxide (ITO) plates at temperature controlled between 120 and 160 °C. Thickness of film was controlled between 60 to 100 μm using a Teflon spacer. Appropriate composite ratio leading to better photorefractive performances was previously investigated [17]. 

### 2.2. Photorefractive Characterization

Electric field in the range of 0 to 45 V μm^−1^ was applied to the sample film. Degenerate four wave mixing (DFWM) technique with He-Ne laser [18] was used to evaluate diffraction efficiency and its response speed. s-Polarized beam was used for interference beam, and p-polarized probe beam for diffraction measurement. Two beam coupling (2BC) measurement with He-Ne laser [18] was performed to evaluate asymmetric energy transfer which is unique feature of photorefractive effect due to phase shift of refractive index modulation to the optical interference pattern. He-Ne laser (3 mW, 1.5 W cm^−2^) is used. Glass transition temperature of sample film is determined by a differential scanning calorimetry (DSC) at a heating rate of 10 °C in nitrogen atmosphere.

### 2.3. Schematic Apparatus for Holographic Imaging

**Figure 2:**
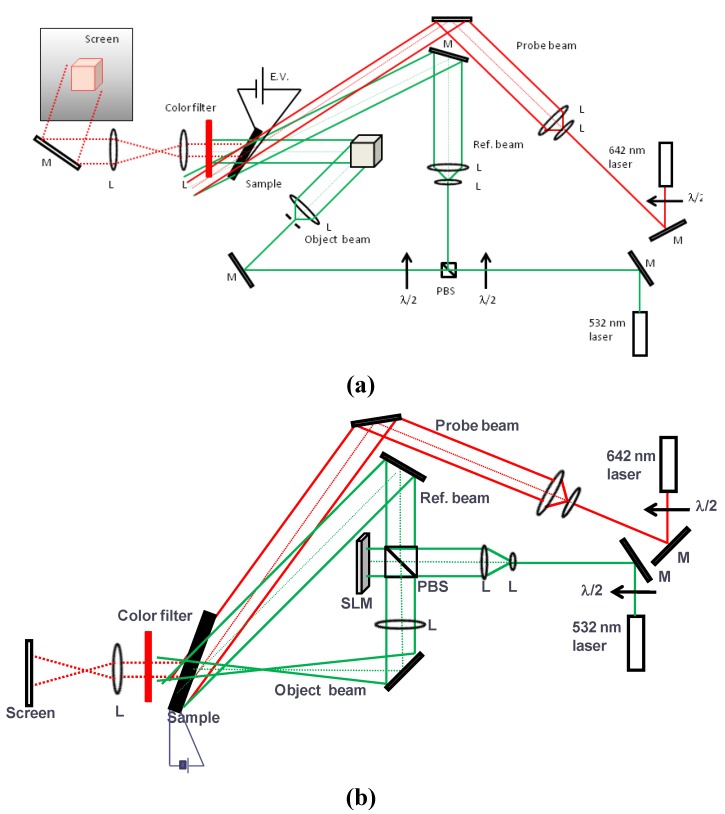
(**a**) Experimental setup for dynamic holographic imaging system for real object; (**b**) and for spatial light modulator (SLM). In transmission, two writing beams (object and reference beams) are incident from the same side. To observe the images, a probe beam is made to forward-propagate with a reference beam. A diffracted image (red line) passes through a color filter and is projected on a screen.

Schematic apparatus for the updatable real time holographic imaging of object is illustrated in Figure 2. Figure 2(a) shows the setup for real object. In this case, two laser sources were used; one is for recording and the other for reconstruction. Beam from a laser source (Spectra Physics, 300 mW at 532 nm) is split off by a polarized beam splitter: One s-polarized beam is spread out with a combination of an objective lens (40×) and a plano-convex lens, which is used for object beam. Another s-polarized beam also spread out is used as a reference beam to interfere with the reflected beam from the object on the PR sample film. Intensity ratio between object and reference beams is controlled with a half wavelength wave plate in front of a polarized beam splitter. Intensity of object beam is 10 mW and that of reference one is 40 mW cm^−2^. Recorded hologram is simultaneously reconstructed by p-polarized probe beam at 642 nm and projected on a white screen. Laser source is an Omicron-Laserage semiconductor laser PhoxX 642 (140 mW at 642 nm). Figure 2b shows a setup for the object image on a spatial light modulator (SLM, 1920 × 1200 Holoeye LCR-1080). A laser source beam is expanded using a combination of objective lens (10×) and a plano-convex lens. s-Polarized expanded beam split off by a beam splitter work as a reference beam and p-polarized beam transmitted through a beam splitter is reflected on a SLM. Reflected beam work as an object beam. Polarization of a reflected beam from SLM is changed to s-polarization. Object and reference beams are interfered on a PR polymer composite and recorded hologram is simultaneously reconstructed by a probe beam and projected on a white screen. Active area size of PR polymer composite is 1.8 cm^2^.

## 3. Results and Discussion 

In a former study [19], we studied the effect of molecular weight of PVCz on the photorefractive performances of diffraction efficiency and its response time. Higher molecular weight of PVCz leads to higher diffraction efficiency and faster response time, because dimer cation sites along longer molecular chain work as effective trap sites for hole charge carriers. In the present case, PVCz with high molecular weight of 370,000 was used. 

The HOMO levels of PVCz (and CzEPA), 7-DCST, and TNF are −5.9 [20], −5.9 [12], and −7.9 eV [21], respectively. These HOMO levels mean that 7-DCST and TNF does not work as a deep trap for hole transport hopping through PVCz and CzEPA.

**Figure 3:**
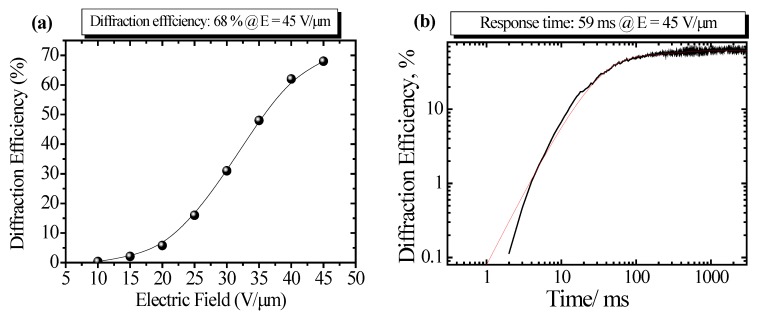
(**a**) Plots of diffraction efficiency as a function of electric field. The sample: polymer composite of PVCz/7-DCST/CzEPA/TNF (44/35/20/1 by wt); (**b**) Time response profile of optical diffraction. Red curve is the plots fitted by bi-exponential function of Equation (1).

Diffraction efficiency is an important fundamental aspect for evaluating PR properties of PR polymer composites, because the brightness of the hologram recorded in the PR polymer composite is significantly affected by the diffraction efficiency. Diffraction efficiency is determined using a DFWM method. Diffraction efficiency is plotted as a function of applied electric field in Figure 3a. Diffraction efficiency significantly depends on the applied electric field, and composite film has a high diffraction efficiency of 68% at *E* = 45 V μm^−1^. 

Response speed of optical diffraction is the key parameter for real-time 3D holographic display. Time response profile of optical diffraction for the same PR polymer composite is shown in Figure 3b. Log-log scale is adapted to clarify the initial evaluation of grating buildup. Bi-exponential function of

(1) $$\eta =\eta_{0}{\left\{p\left(1-e^{-t/\tau_{1}}\right)+\left(1-p\right)\left(1-e^{-t/\tau_{2}}\right)\right\}}^{2}$$

is used to evaluate response property, where η_0_ is the steady-state diffraction efficiency, τ_1_ is the fast component of response time, τ_2_ is the slow component of response time, and p is the contribution of fast component. Fitted parameters of τ_1_ = 24 ms, τ_2_ = 319 ms, p = 0.88 were obtained. Weighted average response time of 59 ms is determined. The response time is significantly related to the formation speed of space charge field and orientation of NLO dye. 

**Figure 4:**
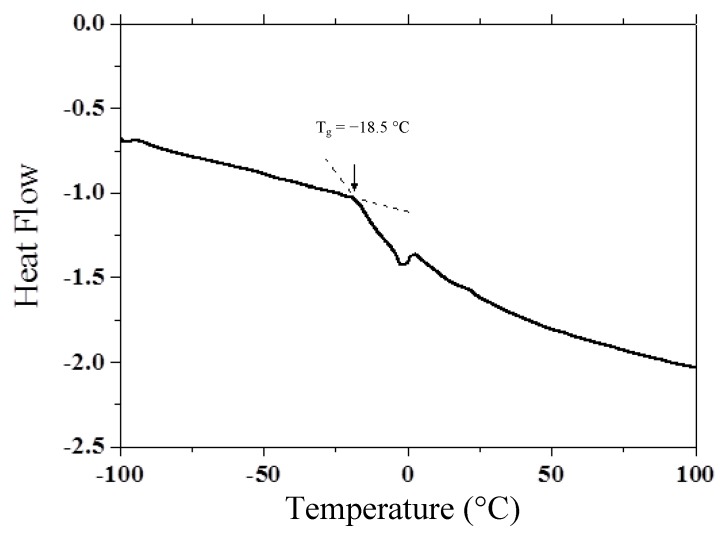
Differential scanning calorimetry (DSC) thermogram of photorefractive (PR) polymer composite.

Orientation of NLO dye, so-called orientational enhancement [22], plays an important role for photorefractive response of diffraction efficiency and optical gain in polymer composite, in addition to normal Pockels effect for photorefractive response. To assist orientation of NLO dye, it is important to keep the glass transition temperature (T_g_) low enough to easily rotate NLO dye in the matrix. T_g_ can be controlled by adjusting content of plasticizer. DSC thermogram of composite is shown in Figure 4. T_g_ of the composite was −18.5 °C.

**Figure 5:**
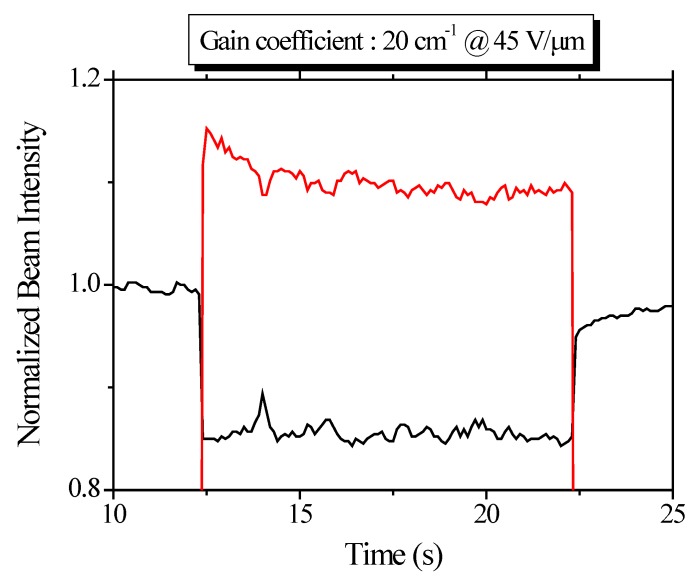
Asymmetric energy transfer measured by two beam coupling (2BC) technique. Beam intensity on y-axis is normalized by the intensity before two beam coupling measurement.

Optical gain caused by the asymmetric energy transfer is the unique feature of photorefractive response of materials. Figure 5 shows the result of two beam coupling of composite. Optical gain of 20 cm^−1^ is measured at 45 V μm^−1^.

**Figure 6:**
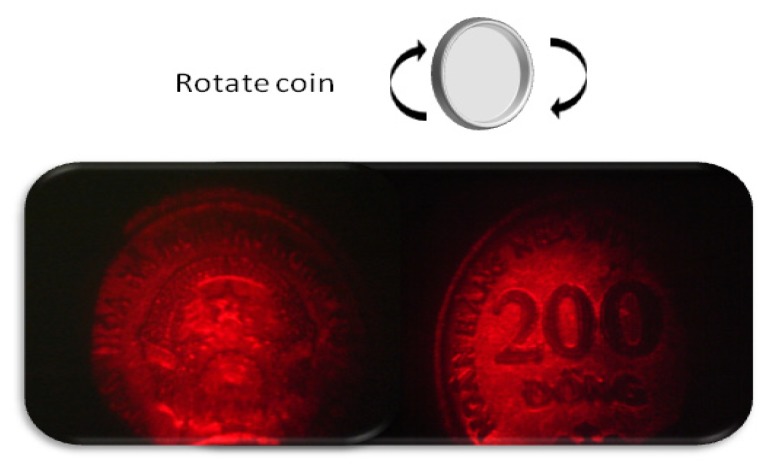
Hologram images of head and tail of coin.

Hologram image of coin was recorded in the same PR polymer composite by applying at 45 V μm^−1^ and simultaneously reconstructed using an apparatus shown in Figure 2a. Since the external intersectional angle between object beam and reference beams is 26°, grating periodicity is 2 μm. Thus lateral resolution is the same as the grating periodicity of 2 μm. Figure 6 is the holograms of head and tail of coin recorded in the PR polymer composite and reconstructed by red probe beam. Hologram of head was first recorded in the PR polymer composite and reconstructed by a red probe beam. Then after rotation of coin, hologram of tail of coin was recorded in the same PR polymer composite and reconstructed by a red probe beam. Hologram from PR polymer composite reproduced the detail of the coins. Updatable holographic images are recorded on the same PR polymer composite. 

**Figure 7:**
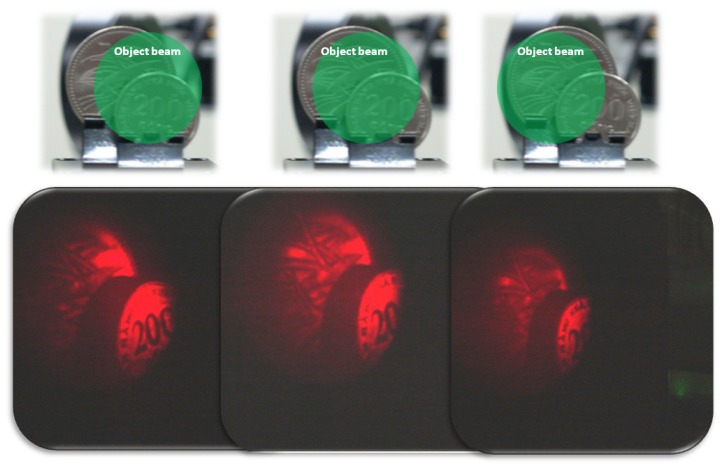
The top shows the photograph of the object. From left to right, coin moves laterally, corresponding hologram was reconstructed.

Quickly updatable hologram images are shown in Figure 7. The top shows the photograph of the object. The bottom shows the reconstructed hologram image after being recorded in the PR polymer composite. Object beam reflected from the coin surface is recorded in the PR polymer composite applying an electric field of 45 V μm^−1^. Hologram is recorded by the interference of reflected object and reference beams and simultaneously reconstructed by a red probe beam. Then after sliding the position of object beam by moving the stage on which coins are standing, the second hologram is recorded and simultaneously reconstructed. Furthermore third hologram is recorded and simultaneously reconstructed. Three holograms of three different surface positions of coins are recorded in turn at the same position of PR polymer composite and simultaneously reconstructed in turn. PR polymer film can record the object image holograms and promptly reconstruct it. All holographic images appeared and disappeared within 1 s when the reference beams was on or off, respectively.

**Figure 8:**
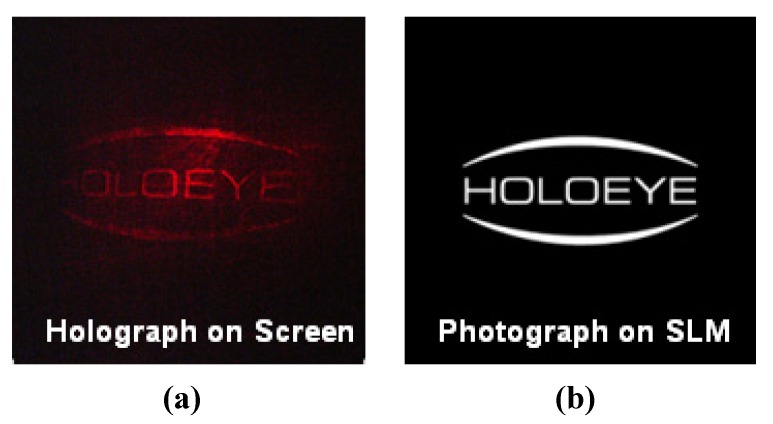
(**a**) Holographic image on screen; and (**b**) original image on SLM.

Next, instead of using a real object, an object image on a spatial light modulator (SLM) which is produced by the computer is used as an object for the hologram. SLM is convenient because it provides both amplitude modulation and phase modulation modes. Reflected object beam from a 1920 × 1200 Holoeye LCR-1080 SLM interfered with reference beam on PR polymer composite to record a hologram and hologram image is simultaneously reconstructed by a red probe beam, whose schematic diagram is shown in Figure 2b. Figure 8 shows the reconstructed holographic image (left in figure) of original image (right in figure) generated by a computer. It is clearly reconstructed. 

The photorefractive polymer features flexibility, transparency, and ease of processing with large area size. Thus, we believe that the photorefractive polymer composite is one of the promising devices for future holographic imaging and display. 

## 4. Conclusions 

We have successfully demonstrated a quickly updatable real-time holographic display using a PR polymer composite based on PVCz with low T_g_. Holograms of object coins are clearly recorded in the PR polymer composite and simultaneously reconstructed by a probe beam. A computer generated image is recorded using the holographic interference technique and a holographic image is simultaneously reconstructed by a probe beam.

## References

[1] Gabor D. (1948). A new microscope principle. Nature.

[2] Ducharme S., Scott J.C., Twieg R.J., Moerner W.E. (1991). Observation of the photorefractive effect in a polymer. Phys. Rev. Lett..

[3] Ostroverkhova O., Moerner W.E. (2004). Organic photorefractives: mechanism, materials and applications. Chem. Rev..

[4] Meerholz K., Volodin B.L., Kippelen B., Peyghambarian N. (1994). A photorefractive polymer with high optical gain and diffraction efficiency near 100%. Nature.

[5] Kippelen B., Meerholz K., Peyghambarian N., Nalwa H.S., Miyata S. (1996). Nonlinear Optics of Organic Molecules and Polymers.

[6] Tay S., Blanche P.A., Voorakaranam R., Tune A.V., Lin W., Rokutanda S., Gu T., Flores D., Wang P., Li G., Hilaire P.S., Thomas J., Norwood R.A., Yamamoto M., Peyghambarian N. (2008). An updatable holographic three-dimensional display. Nature.

[7] Blanche P.A., Bablumian A., Voorakaranam R., Christenson C., Lin W., Gu T., Flores D., Wang P., Hsieh W.Y., Kathaperumal M., Rachwal B., Siddiqui O., Thomas J., Norwood R.A., Yamamoto M., Peyghambarian N. (2010). Holographic three-dimensional telepresence using large-area photorefractive polymer. Nature.

[8] Peyghambarian N., Blanche P.A., Bablumyan A., Yamamoto M., Kawabe Y., Kawase M. (2011). Large area photorefractive polymers for updatable holographic 3D display. Polymer Photonics, and Novel Optical Technologies.

[9] Tsutsumi N., Kinashi K., Sakai W., Kawabe Y., Kawase M. (2011). Strategy for high performance photorefractive polymer composites. Polymer Photonics and Novel Optical Technologies.

[10] Tsutsumi N., Kinashi K., Sakai W., Nishide J., Kawabe Y., Sasabe H. (2012). Real-time three-dimensional holographic display using a monolithic organic compound dispersed film. Opt. Mater. Express.

[11] Koeber S., Salvador M., Meerholz K. (2011). Organic photorefractive materials and applications. Adv. Mater..

[12] Thomas J., Christenson C.W., Blanche P.A., Yamamoto M., Norwood R.A., Peyghambarian N. (2011). Photoconducting polymers for photorefractive 3D display applications. Chem. Mater..

[13] Christenson C.W., Thomas J., Blanche P.A., Voorakaranam R., Norwood R.A., Yamamoto M., Peyghambarian N. (2010). Grating dynamics in a photorefractive polymer with Alq(3) electron traps. Opt. Express.

[14] Christenson C.W., Greenlee C., Lynn B., Thomas J., Blanche P.A., Voorakaranam R., Hilaire P., LaComb L.J., Norwood R.A., Yamamoto M., Peyghambarian N. (2011). Interdigitated coplanar electrodes for enhanced sensitivity. Opt. Lett..

[15] Giang H.N., Kinashi K., Sakai W., Tsutsumi N. (2012). Photorefractive composite based on monolithic polymer. Macromol. Chem. Phys..

[16] Tsujimura S., Kinashi K., Sakai W., Tsutsumi N. (2012). High-speed photorefractive response capability in triphenylamine polymer-based composites. Appl. Phys. Express.

[17] Tsutsumi N., Dohi A., Nonomura A., Sakai W. (2011). Enhanced performances of photorefractive poly(*N*-vinyl carbazole) composites. J. Polym. Sci. Part B Polym. Phys..

[18] Tsutsumi N., Murao T., Sakai W. (2005). Photorefractive reponse of polymeric composites with pendant triphenylamine moiety. Macromolecules.

[19] Tsutsumi N., Kasaba H. (2008). Effect of molecular weightt of poly(N-vinyl carbazole) on photorefractive performances. J. Appl. Phys..

[20] Kippelen B., Blanche P.A., Schülzgen A., Fuentes-Hernandez C., Ramos-Ortiz G., Wang J.F., Peyghambarian N., Marder S.R., Leclercq A., Beljonne D., Bredas J.L. (2002). Photorefractive polymers with non-destructive readout. Adv. Funct. Mater..

[21] Im C., Emelianova E.V., Bässler H., Spreitzer H., Becker H. (2002). Intrinsic and extrinsic charge carrier photogeneration in phenyl-substituted polyphenylenevinylene-trinitrofluorenone blend systems. J. Chem. Phys..

[22] Moerner W.E., Silence S.M., Hache F., Bjorklund G.C. (1994). Orientationally enhanced photorefractive effect in polymers. J. Opt. Soc. Am. B.

